
JOURNAL INFORMATION
==============================
NLM Title Abbreviation: J Mol Cell Biol
Journal ID: J Mol Cell Biol
NLM Journal ID: 101503669
Journal ID: jmcb

Journal of Molecular Cell Biology

ISSN: 1674-2788
EISSN: 1759-4685

ARTICLE INFORMATION
==============================
PMCID: PMC8344664
PMID: 34165565
DOI: 10.1093/jmcb/mjab037
Article ID: mjab037
Article version: 1
Subjects: Application Note, AcademicSubjects/SCI01180

Development of highly sensitive and rapid antigen detection assay for diagnosis of COVID-19 utilizing optical waveguide immunosensor

Funabashi Rikako 1
Miyakawa Kei 1
Yamaoka Yutaro 1 2
Yoshimura Seiko 3
Yamane Satoshi 3
Jeremiah Sundararaj Stanleyraj 1
Shimizu Kohei 4
Ozawa Hiroki 4
Kawakami Chiharu 4
Usuku Shuzo 4
Tanaka Nobuko 4
Yamazaki Etsuko 5
Kimura Hirokazu 6
Hasegawa Hideki 7
Ryo Akihide 1
1 Department of Microbiology, Yokohama City University Graduate School of Medicine , Kanagawa, Japan
2 Life Science Laboratory, Technology and Development Division, Kanto Chemical Co., Inc ., Kanagawa, Japan
3 Primary Care Testing Solution Development Department, Canon Medical Systems Corporation , Tochigi, Japan
4 Yokohama City Institute of Public Health , Kanagawa, Japan
5 Clinical Laboratory Department, Yokohama City University Hospital , Kanagawa, Japan
6 Department of Health Science, Gunma Paz University Graduate School , Gunma, Japan
7 Influenza Research Center, National Institute of Infectious Diseases , Tokyo, Japan
Correspondence to: Akihide Ryo, E-mail: aryo@yokohama-cu.ac.jp
Electronic publication date: 2021 Jun 24
Electronic Location ID: mjab037
Received 2021 Jan 8; Revised 2021 May 1; Accepted 2021 May 12
Copyright: © The Author(s) 2021. Published by Oxford University Press on behalf of Journal of Molecular Cell Biology, IBCB, SIBS, CAS.
Copyright year: 2021
License: This is an Open Access article distributed under the terms of the Creative Commons Attribution License (http://creativecommons.org/licenses/by/4.0/), which permits unrestricted reuse, distribution, and reproduction in any medium, provided the original work is properly cited.
License URL: https://creativecommons.org/licenses/by/4.0/

Page count: 18
edited-state: accepted-manuscript
article-lifecycle: PAP

==============================

Rikako Funabashi[1], Kei Miyakawa[1], Yutaro Yamaoka[1,2], Seiko Yoshimura[3], Satoshi Yamane[3], Sundararaj Stanleyraj Jeremiah[1], Kohei Shimizu[4], Hiroki Ozawa[4], Chiharu Kawakami[4], Shuzo Usuku[4], Nobuko Tanaka[4], Etsuko Yamazaki[5], Hirokazu Kimura[6], Hideki Hasegawa[7], and Akihide Ryo[1,*]

Ever since its outbreak, the COVID-19 pandemic caused by SARS-CoV-2 has been spreading rapidly causing a global health crisis. Accurate diagnosis and instituting appropriate intervention to relevant individuals are essential not only to slow down the spread of this pandemic but also to manage our resources efficiently. To date, testing for SARS-CoV-2 infection mostly relies on reverse transcription quantitative polymerase chain reaction (RT-qPCR) on a nasopharyngeal or saliva specimen and remains the gold standard for diagnosis as it exhibits high sensitivity and specificity. However, RT-qPCR requires expensive equipment, and the centralized testing format practically requires ~24 h to obtain the results warranting more than one visit to the healthcare facility (Brendish et al., 2020). Moreover, a unique drawback that the RT-qPCR faces in COVID-19 diagnosis is that it cannot specifically distinguish between the infectious viruses shed from the respiratory tract and the persisting non-transmissible dead viruses or genetic fragments (Cento et al., 2020).

Rapid antigen detection tests (Ag-RDTs) for SARS-CoV-2 directly detect the viral proteins in respiratory specimen and offer multiple benefits in comparison to RT-qPCR, such as shorter turnaround times, lower cost, decentralized point-of-care testing (POCT), and ease of use (Li and Li, 2021). However, most of the currently available Ag-RDTs lose out to RT-qPCR in terms of sensitivity. This could be due to either the inherent low sensitivity of the assay platform or the apparent reduction in sensitivity when pitched against the RT-qPCR later in course of the infection. Ag-RDTs may also show cross reactivity to other related viruses based on the quality of the detector antibodies used.

In the present study, we improved the sensitivity of SARS-CoV-2 Ag-RDT by utilizing the optical waveguide-based biosensor technology (Uematsu et al., 2016). We further abolished the problem of cross reactivity by using a pair of highly specific monoclonal antibodies (mAbs) targeting SARS-CoV-2 nucleocapsid protein (NP) antigen (Yamaoka et al., 2021b). Thus, the developed Rapiim SARS-CoV-2-N assay reported in this study possessed a detection limit of $9.3\times10^4$ copies/ml and exhibited no cross reactivity with related viruses. This specific and sensitive assay has a high potential for COVID-19 diagnosis as well as for rapid identification of transmission competent individuals.

Rapiim SARS-CoV-2-N was developed based on antigen-sandwich principle using a pair of mAbs (capture antibody conjugated with light scattering particle and detector antibody immobilized on the surface of an optical waveguide film) that specifically detect SARS-CoV-2 NP antigen while binding to different epitopes of the antigen without any spatial interference (Figure 1A). Upon addition of the sample to the processing solution, the capture antibody conjugate binds to the SARS-CoV-2 NP antigen to form larger immune complexes. This solution is added to the cartridge where the detector antibody binds to the incoming immune complexes near the waveguide film. In the analyzer, incident light on the waveguide undergoes total internal reflection at the surface of the waveguide and is emitted out as the outgoing light. During this process, some of the light seeps out of the surface of the film as the evanescent light, which is scattered proportional to the number of immune complexes present on the surface. Evanescent light scattering attenuates the intensity of the outgoing light, which is detected by the optical sensor. The light attenuation rate is analyzed with calculation algorithm within the analyzer and a positive or negative result is displayed on the screen (Figure 1A). With this technique, it is possible to obtain non-subjective, reliable results in a single step as early as 4–15 min.

To check for cross reactivity, we tested viral fluids from 19 respiratory virus particles and 6 recombinant antigens of related coronaviruses in Rapiim SARS-CoV-2-N and found that none of the viruses or recombinant antigens were flagged positive, denoting the exclusive specificity of the test for SARS-CoV-2 (Figure 1B; Supplementary Table S1). To identify the minimum detection sensitivity of the kit in detecting viable viruses, we used serial dilutions of culture supernatants of two differently isolated SARS-CoV-2 namely, SARS-CoV-2 JPN/TY/WK-521 (accession ID: LC522975.1), isolated at the National Institute of Infectious Diseases in January 2020, and SARS-CoV-2 Japan/YCU01/2020 (GISAID accession ID: EPI_ISL_693298), isolated at Yokohama City University in July 2020. In both cases, the limit of detection (LOD) was $9.3 \times 10^4$ copies/ml suggesting that the SARS-CoV-2 antigen from different mutants could be equally detected by Rapiim SARS-CoV-2-N (Supplementary Table S2). Indeed, the epitope regions targeted by mAb1 and mAb2 of the two different virus strains used were identical and conserved from mutations (Supplementary Table S3). Also, Rapiim SARS-CoV-2-N was able to detect SARS-CoV-2 at a much lower LOD than other commercially available rapid antigen detection kits suggesting its superiority over conventional lateral flow immunoassay (LFIA)-based Ag-RDTs (Supplementary Table S4).

We then evaluated Rapiim SARS-CoV-2-N for its minimum detection sensitivity in 58 PCR-positive and 30 PCR-negative nasopharyngeal swabs (Supplementary Table S5). Positive PCR specimens were further classified according to the Ct values, and PCR concordance rates of the two antigen detection assays were compared for high viral titer (Ct <25), medium viral titer (Ct 25–30), and low viral titer (Ct >30) (Supplementary Figure S2). We found that Rapiim SARS-CoV-2-N was able to detect all samples with high and medium viral titers, while it could detect 64.7% (95%CI: 47.8%–78.6%) samples in the low virus titer cohort (Figure 1C). The distribution of tested samples with the virus concentrations in the treated solution was plotted as the vertical axis, showing that the concentration of virus in the specimen that causes Rapiim SARS-CoV-2-N to test negative for true positive specimens is low and remains within a narrow distribution (Figure 1D). This result indicates that Rapiim SARS-CoV-2-N can test positive for true positive specimens with lower virus concentration (Figure 1D). Notably, Rapiim SARS-CoV-2-N was completely concordant in PCR-negative specimens having 100% specificity (Figure 1C).

We examined the relationship between the numerical value of the attenuation rate of the detected light signal and the RNA copy number calculated by RT-qPCR in nasopharyngeal swabs from 58 PCR-confirmed COVID-19 patients and 30 PCR-negative swabs (Supplementary Table S5). The attenuation value of the detected light signal tended to increase proportionately with the amount of virus and showed a very strong correlation ($R^2 = 0.93$, $P$-value <0.0001) (Supplementary Figure S3). A plateau of saturation was observed when the RNA copy number reached $10^6$ copies, beyond which the attenuation rate was as high as 80%–90% regardless of further increase in the virus amount.

Currently, the United States Food and Drug Administration (FDA) has only approved the Ag-RDTs that use NP as a target region (Nguyen et al., 2020). This can cause the problem of cross reactivity due to the antigenic homology of NP conserved between other coronaviruses. This could be overcome by using N-terminally truncated NP (ΔN-NP), which is highly specific for SARS-CoV-2 and abolishes cross reactivity (Yamaoka et al., 2021a, b). Rapiim SARS-CoV-2-N uses two highly specific antibodies derived from ΔN-NP that exclusively detect SARS-CoV-2. Our study has shown Rapiim SARS-CoV-2-N to possess 100% specificity as all PCR-negative samples were flagged negative. Additionally, the epitope sequences of these two detector antibodies were compared with those of ~8000 SARS-CoV-2 strains isolated so far and were found to be 99.75% and 99.41% identical, respectively, suggesting that these antibodies can detect the mutational variants of SARS-CoV-2 (Yamaoka et al., 2021b).

COVID-19 is a biphasic illness with an initial viral phase followed by an inflammatory phase. Transmissibility of SARS-CoV-2 begins shortly after infection in the asymptomatic period, peaks around the time of symptoms onset, and rapidly decreases 7 days thereafter (Stanleyraj et al., 2021). This is reflected by parallel changes in the viral load in the respiratory tract of infected individuals over time, which reaches its highest level around the time of symptoms onset and declines linearly thereafter (Sethuraman et al., 2020). Most of the currently available Ag-RDTs are based on LFIA platform, which is a rapid test method using antigen–antibody reaction as a detection principle and is based on an immunoassay performed by the unidirectional flow of sample over a test strip. However, it has the inherent drawback of lesser sensitivity. Lower sensitivity also indicates that the test may miss out detecting positive patients in the early stages of infection when used for screening. Among the various novel methods investigated to improve rapid tests, one is optical waveguide biosensors (Uematsu et al., 2016). We have developed Rapiim SARS-CoV-2-N utilizing this technology and have shown that it achieves a lower LOD compared to the conventional LFIAs. We have shown that even in swab specimens with a low amount of virus, i.e. over $10^4$ copies, which give relatively lower cycle threshold (Ct <30) of RT-qPCR, can almost always be tested positive by Rapiim SARS-CoV-2-N. Hence, it can be expected to reduce the number of false negatives while screening patients with low viral load in the early stages of infection. Rapiim SARS-CoV-2-N also abolishes errors of subjective interpretation such as ‘faint bands’ faced in LFIAs. Although we did not encounter the problem of false positivity due to sample viscosity in any of the 88 clinical samples tested, this should be evaluated on larger scale. Rapiim SARS-CoV-2-N is inexpensive and easy to use, although it requires a dedicated analyzer. Due to all these reasons, Rapiim SARS-CoV-2-N can supplement the molecular diagnosis in order to facilitate healthcare workers to institute timely and appropriate therapeutic or preventive measures for COVID-19.

*[Rapiim is a registered trademark of Canon Medical Systems Corporation. We thank Natsumi Takaira, Kenji Yoshihara, and Kazuo Horikawa for their technical assistance. This work was supported in part by Japan Agency for Medical Research and Development (AMED; JP19fk0108110 and JP20he0522001) and by Health Labour Sciences research grant from The Ministry of Health Labour and Welfare (19HA1003) to A.R. Y.Y. is a current employee of Kanto Chemical Co., Inc. S.Yoshimura and S.Yamane are current employees of Canon Medical Systems Corporation. R.F. performed the research, analyzed the data, and wrote the manuscript; K.M. and Y.Y. designed the study and analyzed the data; S.Yoshimura and S.Yamane performed the research and analyzed the data; S.S.J. analyzed the data and wrote the manuscript; K.S., H.O., C.K., S.U., N.T., and E.Y. contributed specimens; H.K. and H.H. analyzed the data; A.R. directed the research, analyzed the data, and wrote the manuscript.]*

**Figure legend**

**Figure 1** Development of highly sensitive and rapid antigen detection assay for diagnosis of COVID-19. (**A**) Schematic illustration of the principle of Rapiim SARS-CoV-2-N. The processing solution contains light scattering particles conjugated with a highly specific anti-SARS-CoV-2 NP antibody (capture antibody), while the cartridge contains an optical waveguide thin film embedded with another highly specific anti-SARS-CoV-2 NP antibody (detector antibody). Upon addition of a positive swab sample, the two antibodies capture SARS-CoV-2 antigen to form larger immune complexes (light scattering particle–capture antibody–antigen–detector antibody) on the surface of the optical waveguide thin film. In the analyzer, the scattering particles attenuate the outgoing light proportional to the number of immune complexes formed. The attenuated outgoing light is detected by the optical sensor and interpreted by the algorithm to finally provide a positive or negative result on the display screen. (**B**) Cross reactivity test. Either the whole virus or recombinant NP antigen of the mentioned viruses was tested by the Rapiim SARS-CoV-2-N assay. (**C**) Performance of Rapiim SARS-CoV-2-N in detecting PCR-positive samples stratified by viral load. Positive PCR specimens were further classified according to the Ct values, and PCR concordance rates of the Rapiim SARS-CoV-2-N assay were compared for high viral titer (Ct <25), medium viral titer (Ct 25–30), and low viral titer (Ct >30). (**D**) Concentration distribution of viral load-stratified PCR-positive clinical specimens detected by the Rapiim SARS-CoV-2-N assay. Distribution of the results of the Rapiim SARS-CoV-2-N assay against the virus concentration in the vertical axis. PCR-positive samples with high (Ct <25) or medium (Ct 25–30) viral titer are flagged positive while those of low viral titer (Ct >30) are invariably flagged negative by Rapiim SARS-CoV-2-N.

## Figure 1

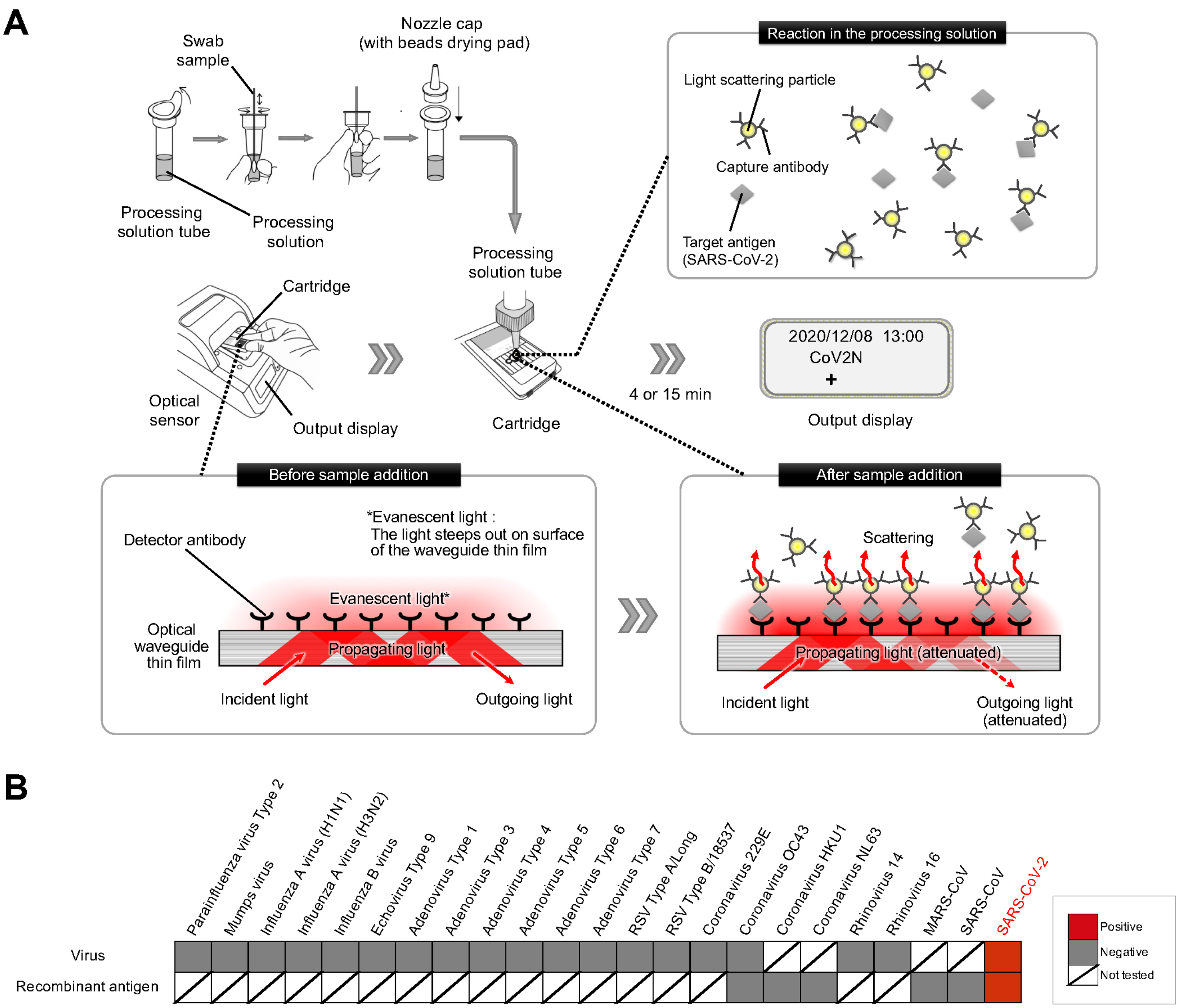

**C**

| | Rapiim SARS-CoV-2-N | | |
|---|---|---|---|
| PCR-positive ($n$ = 58) | Positive | Negative | Sensitivity (95% CI) |
| Ct value | | | |
| < 25 | 5 | 0 | 100.0% (51.1–100.0) |
| 25–30 | 19 | 0 | 100.0% (80.2–100.0) |
| > 30 | 22 | 12 | 64.7% (47.8–78.6) |

| | Positive | Negative | Specificity (95% CI) |
|---|---|---|---|
| PCR-negative ($n$ = 30) | 0 | 30 | 100.0% (86.5–100.0) |

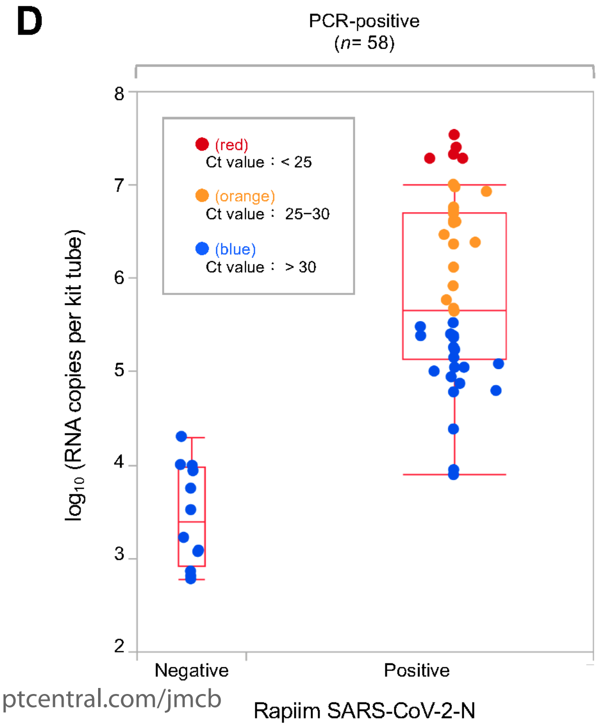

**Supplementary material**

## Development of highly sensitive and rapid antigen detection assay for diagnosis of COVID-19 utilizing optical waveguide immunosensor

Rikako Funabashi[1], Kei Miyakawa[1], Yutaro Yamaoka[1,2], Seiko Yoshimura[3], Satoshi Yamane[3], Sundararaj Stanleyraj Jeremiah[1], Kohei Shimizu[4], Hiroki Ozawa[4], Chiharu Kawakami[4], Shuzo Usuku[4], Nobuko Tanaka[4], Etsuko Yamazaki[5], Hirokazu Kimura[6], Hideki Hasegawa[7], and Akihide Ryo[1,*]

[1] Department of Microbiology, Yokohama City University Graduate School of Medicine, Kanagawa, Japan

[2] Life Science Laboratory, Technology and Development Division, Kanto Chemical Co., Inc., Kanagawa, Japan

[3] Primary Care Testing Solution Development Department, Canon Medical Systems Corporation, Tochigi, Japan

[4] Yokohama City Institute of Public Health, Kanagawa, Japan

[5] Clinical Laboratory Department, Yokohama City University Hospital, Kanagawa, Japan

[6] Department of Health Science, Gunma Paz University Graduate School, Gunma, Japan

[7] Influenza Research Center, National Institute of Infectious Diseases, Tokyo, Japan

[*] Correspondence to:

Akihide Ryo, M.D., Ph.D.

Department of Microbiology, Yokohama City University School of Medicine

3-9 Fuku-ura, Kanazawa, Yokohama, Kanagawa 236-0004, Japan

Tel: +81-45-787-2602

E-mail: aryo@yokohama-cu.ac.jp

**Supplementary Figure S1**

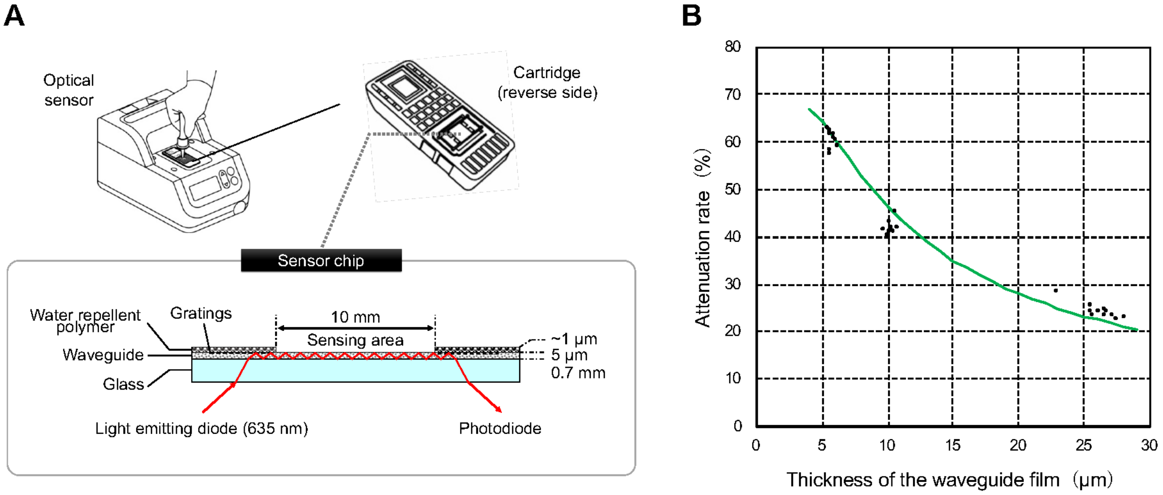

**Supplementary Figure S1. Overview of Rapiim SARS-CoV-2-N**

**(A) Overview of the sensor chip of Rapiim SARS-CoV-2-N**

The sensor chip of the cartridge is formed on a glass substrate with an acrylic resin optical waveguide film, a diffraction grating at both ends, and a water-repellent resin film to protect the diffraction grating. The LED light (635 nm) incident on one of the diffraction gratings is diffracted by the grating and propagates through the optical waveguide film by total reflection.

**(B) Correlation of sensitivity of output signal with the thickness of the waveguide film**

The greater the number of times the propagating light is reflected, the greater the attenuation, and the more sensitive the optical sensor can be formed. The number of reflections can be calculated as L /d tanθ from the diffraction angle θ - determined by the angle of incidence from the LED, the glass refractive index, and the grating pitch (1040 nm), the propagation light path length L (10 mm), and the waveguide film thickness d (5 µm). As the waveguide thickness is reduced, the variation of the attenuation rate is 25 µm: 23.26, 10 µm: 46.41, and 5 µm: 64.23, respectively. By decreasing the waveguide thickness from 25 µm to 5 µm, the sensitivity is increased by a factor of 2.8.

### Supplementary Figure S2

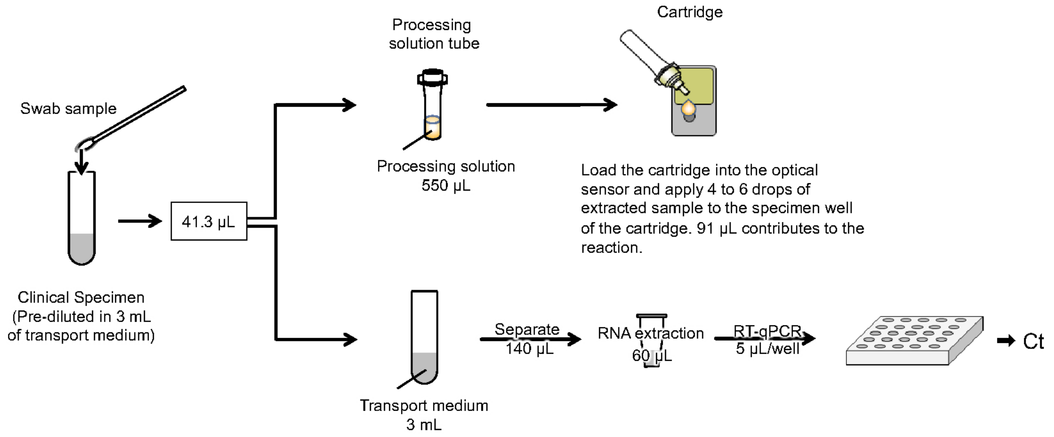

**Supplementary Figure S2. Calculating the Ct value**

41.3 μL of clinical samples (pre-diluted in 3 mL of transport medium) was added to 550 μL of processing solution and was subjected to Rapiim SARS-CoV-2-N assay. The same amount of clinical samples (pre-diluted in 3 mL of transport medium) was added to 3 mL of fresh transport medium and was used as swab samples in this assay, on which RT-qPCR was performed. This algorithm provides precise Ct values for comparing the two tests.

**Supplementary Figure S3**

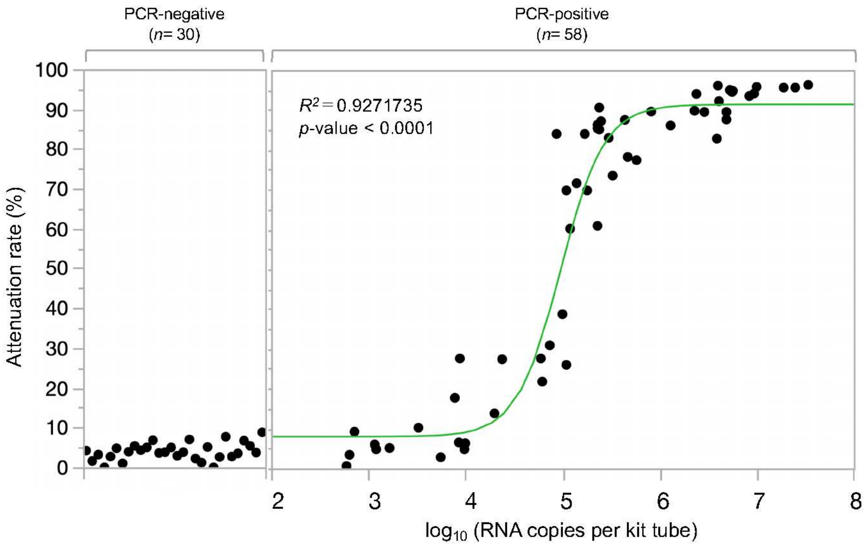

**Supplementary Figure S3. Relationship between attenuation rate values and RT-qPCR RNA Copy Numbers**

The attenuation value of the detected light signal plotted against the amount of virus ($R^2$ = 0.93, *p*-value < 0.0001).

**Supplementary Table S1**

| Virus | Titer ($TCID_{50}$/ml) | Result |
|---|---|---|
| Parainfluenza virus Type 2 | $1.0 \times 10^5$ | Negative |
| Mumps virus | $2.0 \times 10^5$ | Negative |
| Influenza A virus (H1N1) | $1.0 \times 10^6$ | Negative |
| Influenza A virus (H3N2) | $1.0 \times 10^5$ | Negative |
| Influenza B virus | $2.0 \times 10^5$ | Negative |
| Echovirus Type 9 | $1.0 \times 10^9$ | Negative |
| Adenovirus Type 1 | $2.0 \times 10^8$ | Negative |
| Adenovirus Type 3 | $1.0 \times 10^9$ | Negative |
| Adenovirus Type 4 | $2.0 \times 10^7$ | Negative |
| Adenovirus Type 5 | $2.0 \times 10^7$ | Negative |
| Adenovirus Type 6 | $2.0 \times 10^{10}$ | Negative |
| Adenovirus Type 7 | $2.0 \times 10^7$ | Negative |
| RSV Type A/Long | $1.0 \times 10^5$ | Negative |
| RSV Type B/18537 | $1.0 \times 10^5$ | Negative |
| Rhinovirus 14 | $1.1 \times 10^6$ | Negative |
| Rhinovirus 16 | $3.6 \times 10^5$ | Negative |
| | Concentration (copies/ml) | |
| Coronavirus 229E | $1.0 \times 10^7$ | Negative |
| Coronavirus OC43 | $1.0 \times 10^7$ | Negative |
| SARS-CoV-2 | $9.3 \times 10^4$ | Positive |

| Recombinant antigen | Concentration (pg/ml) | Result |
|---|---|---|
| MERS-CoV NP | $2.5 \times 10^7$ | Negative |
| SARS-CoV NP | $2.5 \times 10^7$ | Negative |
| HCoV-HKU1 NP | $2.5 \times 10^7$ | Negative |
| HCoV-OC43 NP | $2.5 \times 10^7$ | Negative |
| HCoV-NL63 NP | $2.5 \times 10^7$ | Negative |
| SARS-CoV-2 NP | $0.7 \times 10^1$ | Positive |

**Supplementary Table S2**

| | SARS-CoV-2 (JPN/TY/WK-521, Accession ID:LC522975.1) | | | SARS-CoV-2 (Japan/YCU01/2020, Accession ID: EPI_ISL_693298) | | |
|---|---|---|---|---|---|---|
| Final concentration (copies/ml) | $1.4 \times 10^5$ | $9.3 \times 10^4$ | $7.0 \times 10^4$ | $1.4 \times 10^5$ | $9.3 \times 10^4$ | $7.0 \times 10^4$ |
| Result | + | + + + (n=3) | + - - (n=3) | + | + + + (n=3) | - - - (n=3) |

**Supplementary Table S3**

| Epitope of detector antibody | | | | |
|---|---|---|---|---|
| Accession ID | | | Sequence | |
| NC_045512 | Reference genome | 335 | GAIKLDDKDPNFKD | 348 |
| LC522975.1 | SARS-CoV-2 (JPN/TY/WK-521) | 335 | GAIKLDDKDPNFKD | 348 |
| EPI_ISL_693298 | SARS-CoV-2 (Japan/YCU01/2020) | 335 | GAIKLDDKDPNFKD | 348 |

| Epitope of capture antibody | | | | |
|---|---|---|---|---|
| Accession ID | | | Sequence | |
| NC_045512 | Reference genome | 382 | LPQRQKKQQTVTLLPA | 397 |
| LC522975.1 | SARS-CoV-2 (JPN/TY/WK-521) | 382 | LPQRQKKQQTVTLLPA | 397 |
| EPI_ISL_693298 | SARS-CoV-2 (Japan/YCU01/2020) | 382 | LPQRQKKQQTVTLLPA | 397 |

**Supplementary Table S4**

| | SARS-CoV-2 JPN/TY/WK-521 (copies/ml) |
|---|---|
| Rapiim SARS-CoV-2-N (Canon Medical Systems) | $9.3\times10^{4}$ |
| ESPLINE SARS-CoV-2 (Fujirebio) | $3.5\times10^{5}$ |
| QUICK NAVI CoV-2 Ag (Denka) | $1.5\times10^{6}$ |

**Supplementary Table S5**

| | | All | PCR-positive | PCR-negative |
|---|---|---|---|---|
| Total | | 88 | 58 | 30 |
| Sex | Male | 53 | 38 | 15 |
| | Female | 42 | 27 | 15 |
| Age | Median | 32.2 | 31 | 34.5 |
| | Range | 1−88 | 3−88 | 1−88 |
| Ct value | Median | | 31.1 | |
| | < 25 | | 5 | |
| | 25−30 | | 19 | |
| | > 30 | | 34 | |

### Materials and methods

#### Ethics statement

This study was approved by the Institutional Review Board of Yokohama City University (IRB No. B200800106), and the protocols used in the study were approved by the ethics committee.

#### Generation of specific monoclonal antibodies against SARS-CoV-2 NP

N-terminal truncated nucleocapsid protein (ΔN-NP; aa 121−419) devoid of the homologous residues and used it as an immunogen to produce mAbs that are specific for SARS-CoV-2. His-tagged ΔN-NP was successfully expressed using wheat germ extracts and was purified as a soluble protein for immunization to BALB/c mice. After 4 weeks of immunization, lymphocytes were harvested from the immunized mice and were fused with mouse myeloma cells to establish hybridomas designated (Yamaoka et al., 2021b). The epitope of the antibodies used in this kit is listed in Supplementary Table S3.

#### Materials and Design of Rapiim SARS-CoV-2-N

The sensor chip of the cartridge is made of an acrylic resin optical waveguide film on a glass substrate, the gratings at both ends, and a water-repellent resin film to protect the gratings. The LED light (635 nm) incident on one of the diffraction gratings is diffracted and propagates through the optical waveguide film by total reflection. The light is again diffracted by the other diffraction gratings and the emitted light intensity is detected by a photodiode. The surface of the light-scattering particles (1.5 μm in diameter) was coated with a low protein-adsorbing dendritic film. Antibodies were immobilized on the light-scattering particles with carboxyl groups by an amino coupling method using EDC (1-Ethyl-3-(3-dimethylaminopropyl)carbodiimide) and Su-NHS (N-Hydroxysulfosuccinimide-Suc: Sulfosuccinimidyl). Subsequently, blocking was carried out by the action of amino PEG (Polyethylene glycol). When an antigen-mediated sandwich is formed between the light-scattering particles and the surface of the light-waveguide film, where monoclonal antibodies are immobilized respectively, the seeping waves (evanescent waves) originating from the propagating light are scattered by the particles and the propagating light is attenuated.

**Operation Procedure of Rapiim SARS-CoV-2-N**

Once the sample has been prepared, remove the cap from the processing solution tube, add the sample, and press the nozzle cap tightly onto the tube. Load the cartridge into the optical sensor and apply 4 to 6 drops of extracted sample to the specimen well of the cartridge. Slide the lid of the optical sensor toward you to start measurement, and the automatic result will be shown on the display after 4 or 15 minutes.

**Antigen detection tests**

For LOD measurement using recombinant antigen, SARS-CoV2 NP (Kanto Chemical, 38157-96) was serially diluted in 9.6 mM PBS (1% BSA, 0.1% Triton). The minimum detection sensitivity of the recombinant antigen was determined by using the concentration of the Bicinchoninic Acid (BCA) method, which is a common method for protein quantification. For this test, 41.3 μL of recombinant antigen was added to 550 μL of sample solution. The minimum concentration of recombinant antigen was set at 100% of the positive result rate. For LOD measurement using Cultured Virus, viral supernatant was diluted in PBS, and 41.3 μL of cultured virus solution was added to sample treatment solution (550 μL) for this test. The assay was performed by adding 41.3 μL of the cultured virus solution diluted to the minimum concentration judged to be positive to the sample treatment solution (550 μL) (n=3). Based on the results of the above tests, the virus concentration at the maximum dilution at which a positive result was obtained using the cultured virus solution was used as an index of minimum detection sensitivity. For additional comparison, the swabs were also tested at exact same conditions using the previously approved SARS-CoV-2 antigen detecting rapid diagnostic kits, ESPLINE SARS-CoV-2 (Fujirebio, Tokyo, Japan) and QUICK NAVI CoV-2 Ag (Denka, Tokyo, Japan). The test was conducted according to the operation method of the kit.

For LOD measurement using Clinical Specimens, nasopharyngeal swabs used in the administrative test (real-time RT-PCR) and whose concentration (number of cycles and copy number) in real-time RT-PCR was calculated were transported from Yokohama City University Hospital and Yokohama City Institute of Public Health to the Department of Microbiology, Yokohama City University School of Medicine. All specimens were transported in virus transport medium with nasopharyngeal swabs, and the performance comparison test with the real-time RT-PCR method was performed using this kit (Rapiim SARS-CoV-2-N) and the previously approved SARS-CoV-2 antigen. The assay was performed by adding 41.3 μL of the Clinical Specimens (Pre-diluted in 3 mL of transport medium) to the sample treatment solution (550 μL).

**RT-qPCR**

Nasopharyngeal swab specimens from individuals confirmed with SARS-CoV-2 infection by RT-qPCR with N2 primer/probe set targeting the N gene were used for this evaluation (Shirato et al., 2020). Samples were stored in viral transport media at –80 ºC until use. Virus load in samples was estimated from cycle threshold (Ct) value. The study protocol was approved by the IRB of Yokohama City University (B200800106).

**Statistical analysis**

All graphs show box-and-whisker or distribution plots and approximate curves. Statistical significance was assessed by JMP 15 (SAS Institute Inc., Cary, NC, USA).

Supplementary Material

mjab037_Supplementary_Data

