# Supplementary material for: Development of highly sensitive and rapid antigen detection assay for diagnosis of COVID-19 utilizing optical waveguide immunosensor
Source: J Mol Cell Biol. 2021 Jun 24;13(10):763–6. doi: 10.1093/jmcb/mjab037 (PMC8344664; doi:10.1093/jmcb/mjab037)
Supplement: mjab037_Supplementary_Data [file mjab037_supplementary_data.pdf]

Supplementary material

Development of highly sensitive and rapid antigen detection assay for diagnosis of COVID-19 utilizing optical waveguide immunosensor

Rikako Funabashi[1], Kei Miyakawa[1], Yutaro Yamaoka[1,2], Seiko Yoshimura[3], Satoshi Yamane[3], Sundararaj Stanleyraj Jeremiah[1], Kohei Shimizu[4], Hiroki Ozawa[4], Chiharu Kawakami[4], Shuzo Usuku[4], Nobuko Tanaka[4], Etsuko Yamazaki[5], Hirokazu Kimura[6], Hideki Hasegawa[7], and Akihide Ryo[1,*]

[1] Department of Microbiology, Yokohama City University Graduate School of Medicine, Kanagawa, Japan

[2] Life Science Laboratory, Technology and Development Division, Kanto Chemical Co., Inc., Kanagawa, Japan

[3] Primary Care Testing Solution Development Department, Canon Medical Systems Corporation, Tochigi, Japan

[4] Yokohama City Institute of Public Health, Kanagawa, Japan

[5] Clinical Laboratory Department, Yokohama City University Hospital, Kanagawa, Japan

[6] Department of Health Science, Gunma Paz University Graduate School, Gunma, Japan

[7] Influenza Research Center, National Institute of Infectious Diseases, Tokyo, Japan

* Correspondence to:

Akihide Ryo, M.D., Ph.D.

Department of Microbiology, Yokohama City University School of Medicine

3-9 Fuku-ura, Kanazawa, Yokohama, Kanagawa 236-0004, Japan

Tel: +81-45-787-2602

E-mail: aryo@yokohama-cu.ac.jp

Supplementary Figure S1

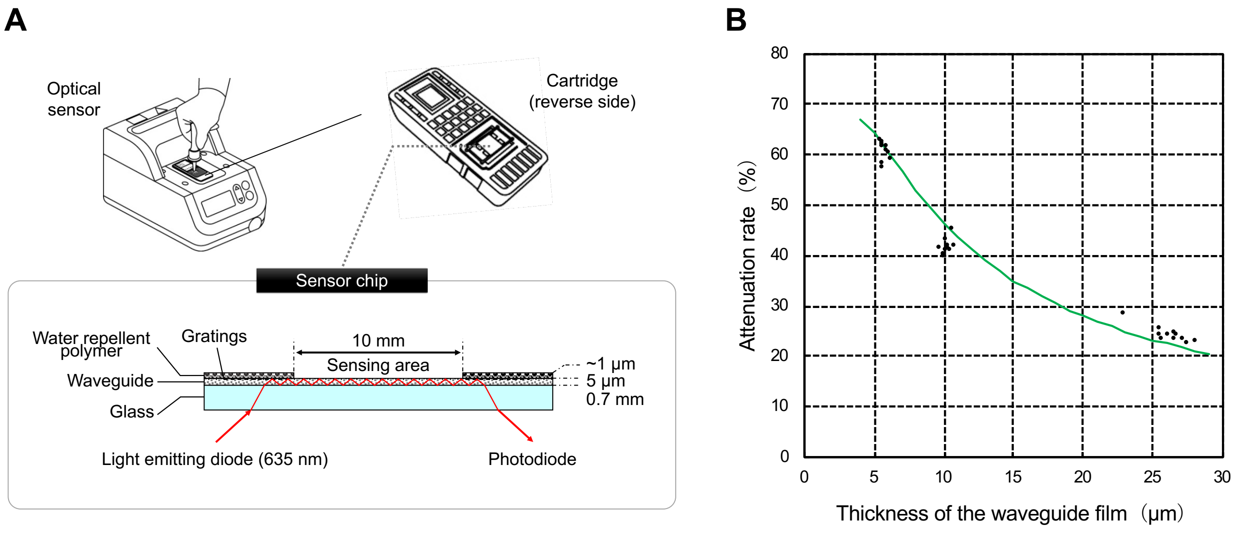

Supplementary Figure S1. Overview of Rapiim SARS-CoV-2-N

(A) Overview of the sensor chip of Rapiim SARS-CoV-2-N

The sensor chip of the cartridge is formed on a glass substrate with an acrylic resin optical waveguide film, a diffraction grating at both ends, and a water-repellent resin film to protect the diffraction grating. The LED light (635 nm) incident on one of the diffraction gratings is diffracted by the grating and propagates through the optical waveguide film by total reflection.

(B) Correlation of sensitivity of output signal with the thickness of the waveguide film

The greater the number of times the propagating light is reflected, the greater the attenuation, and the more sensitive the optical sensor can be formed. The number of reflections can be calculated as L /d tanθ from the diffraction angle θ - determined by the angle of incidence from the LED, the glass refractive index, and the grating pitch (1040 nm), the propagation light path length L (10 mm), and the waveguide film thickness d (5 μm). As the waveguide thickness is reduced, the variation of the attenuation rate is 25 μm: 23.26, 10 μm: 46.41, and 5 μm: 64.23, respectively. By decreasing the waveguide thickness from 25 μm to 5 μm, the sensitivity is increased by a factor of 2.8.

Supplementary Figure S2

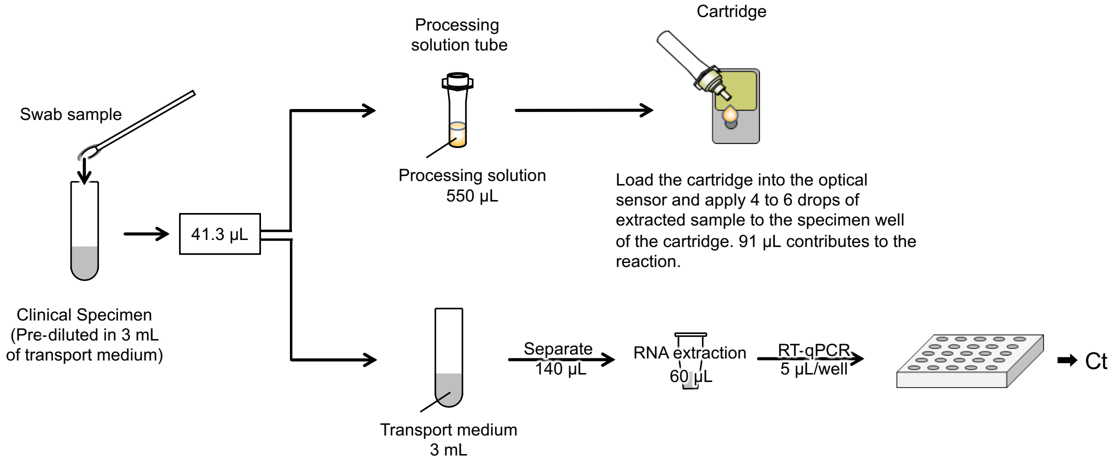

Supplementary Figure S2. Calculating the Ct value

41.3 μL of clinical samples (pre-diluted in 3 mL of transport medium) was added to 550 μL of processing solution and was subjected to Rapiim SARS-CoV-2-N assay. The same amount of clinical samples (pre-diluted in 3 mL of transport medium) was added to 3 mL of fresh transport medium and was used as swab samples in this assay, on which RT-qPCR was performed. This algorithm provides precise Ct values for comparing the two tests.

Supplementary Figure S3

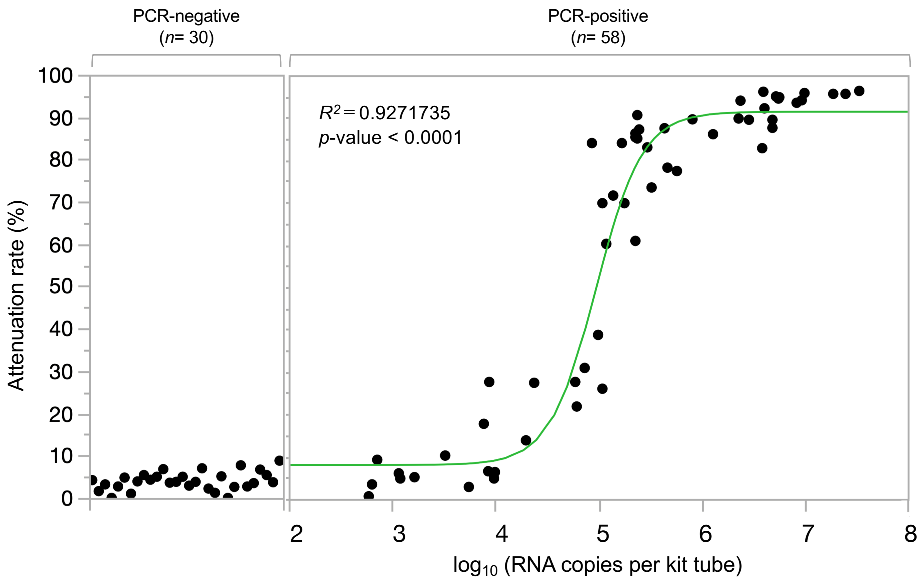

Supplementary Figure S3. Relationship between attenuation rate values and RT-qPCR RNA Copy Numbers

The attenuation value of the detected light signal plotted against the amount of virus (R^2 = 0.93, *p*-value < 0.0001).

Supplementary Table S1

Virus	Titer ($TCID_{50}$/ml)	Result
Parainfluenza virus Type 2	1.0×10^{5}	Negative
Mumps virus	2.0×10^{5}	Negative
Influenza A virus (H1N1)	1.0×10^{6}	Negative
Influenza A virus (H3N2)	1.0×10^{5}	Negative
Influenza B virus	2.0×10^{5}	Negative
Echovirus Type 9	1.0×10^{9}	Negative
Adenovirus Type 1	2.0×10^{8}	Negative
Adenovirus Type 3	1.0×10^{9}	Negative
Adenovirus Type 4	2.0×10^{7}	Negative
Adenovirus Type 5	2.0×10^{7}	Negative
Adenovirus Type 6	2.0×10^{10}	Negative
Adenovirus Type 7	2.0×10^{7}	Negative
RSV Type A/Long	1.0×10^{5}	Negative
RSV Type B/18537	1.0×10^{5}	Negative
Rhinovirus 14	1.1×10^{6}	Negative
Rhinovirus 16	3.6×10^{5}	Negative
	Concentration (copies/ml)	
Coronavirus 229E	1.0×10^{7}	Negative
Coronavirus OC43	1.0×10^{7}	Negative
SARS-CoV-2	9.3×10^{4}	Positive

Recombinant antigen	Concentration (pg/ml)	Result
MERS-CoV NP	2.5×10^{7}	Negative
SARS-CoV NP	2.5×10^{7}	Negative
HCoV-HKU1 NP	2.5×10^{7}	Negative
HCoV-OC43 NP	2.5×10^{7}	Negative
HCoV-NL63 NP	2.5×10^{7}	Negative
SARS-CoV-2 NP	0.7×10^{1}	Positive

Supplementary Table S2

	SARS-CoV-2 (JPN/TY/WK-521, Accession ID:LC522975.1)			SARS-CoV-2 (Japan/YCU01/2020, Accession ID: EPI_ISL_693298)		
Final concentration (copies/ml)	1.4×10^5	9.3×10^4	7.0×10^4	1.4×10^5	9.3×10^4	7.0×10^4
Result	+	+ + + (n=3)	+ - - (n=3)	+	+ + + (n=3)	- - - (n=3)

Supplementary Table S3

Epitope of detector antibody				
Accession ID		Sequence		
NC_045512	Reference genome	335	GAIKLDDKDPNFKD	348
LC522975.1	SARS-CoV-2 (JPN/TY/WK-521)	335	GAIKLDDKDPNFKD	348
EPI_ISL_693298	SARS-CoV-2 (Japan/YCU01/2020)	335	GAIKLDDKDPNFKD	348

Epitope of capture antibody				
Accession ID		Sequence		
NC_045512	Reference genome	382	LPQRQKKQQTVTLLPA	397
LC522975.1	SARS-CoV-2 (JPN/TY/WK-521)	382	LPQRQKKQQTVTLLPA	397
EPI_ISL_693298	SARS-CoV-2 (Japan/YCU01/2020)	382	LPQRQKKQQTVTLLPA	397

Supplementary Table S4

	SARS-CoV-2 JPN/TY/WK-521 (copies/ml)
Rapiim SARS-CoV-2-N (Canon Medical Systems)	9.3×10^{4}
ESPLINE SARS-CoV-2 (Fujirebio)	3.5×10^{5}
QUICK NAVI CoV-2 Ag (Denka)	1.5×10^{6}

Supplementary Table S5

		All	PCR-positive	PCR-negative
Total		88	58	30
Sex	Male	53	38	15
	Female	42	27	15
Age	Median	32.2	31	34.5
	Range	1−88	3−88	1−88
Ct value	Median		31.1	
	< 25		5	
	25−30		19	
	> 30		34	

Materials and methods

Ethics statement

This study was approved by the Institutional Review Board of Yokohama City University (IRB No. B200800106), and the protocols used in the study were approved by the ethics committee.

Generation of specific monoclonal antibodies against SARS-CoV-2 NP

N-terminal truncated nucleocapsid protein (ΔN-NP; aa 121–419) devoid of the homologous residues and used it as an immunogen to produce mAbs that are specific for SARS-CoV-2. His-tagged ΔN-NP was successfully expressed using wheat germ extracts and was purified as a soluble protein for immunization to BALB/c mice. After 4 weeks of immunization, lymphocytes were harvested from the immunized mice and were fused with mouse myeloma cells to establish hybridomas designated (Yamaoka et al., 2021b). The epitope of the antibodies used in this kit is listed in Supplementary Table S3.

Materials and Design of Rapiim SARS-CoV-2-N

The sensor chip of the cartridge is made of an acrylic resin optical waveguide film on a glass substrate, the gratings at both ends, and a water-repellent resin film to protect the gratings. The LED light (635 nm) incident on one of the diffraction gratings is diffracted and propagates through the optical waveguide film by total reflection. The light is again diffracted by the other diffraction gratings and the emitted light intensity is detected by a photodiode. The surface of the light-scattering particles (1.5 μm in diameter) was coated with a low protein-adsorbing dendritic film. Antibodies were immobilized on the light-scattering particles with carboxyl groups by an amino coupling method using EDC (1-Ethyl-3-(3-dimethylaminopropyl)carbodiimide) and Su-NHS (N-Hydroxysulfosuccinimide-Suc: Sulfosuccinimidyl). Subsequently, blocking was carried out by the action of amino PEG (Polyethylene glycol). When an antigen-mediated sandwich is formed between the light-scattering particles and the surface of the light-waveguide film, where monoclonal antibodies are immobilized respectively, the

seeping waves (evanescent waves) originating from the propagating light are scattered by the particles and the propagating light is attenuated.

Operation Procedure of Rapiim SARS-CoV-2-N

Once the sample has been prepared, remove the cap from the processing solution tube, add the sample, and press the nozzle cap tightly onto the tube. Load the cartridge into the optical sensor and apply 4 to 6 drops of extracted sample to the specimen well of the cartridge. Slide the lid of the optical sensor toward you to start measurement, and the automatic result will be shown on the display after 4 or 15 minutes.

Antigen detection tests

For LOD measurement using recombinant antigen, SARS-CoV2 NP (Kanto Chemical, 38157-96) was serially diluted in 9.6 mM PBS (1% BSA, 0.1% Triton). The minimum detection sensitivity of the recombinant antigen was determined by using the concentration of the Bicinchoninic Acid (BCA) method, which is a common method for protein quantification. For this test, 41.3 µL of recombinant antigen was added to 550 µL of sample solution. The minimum concentration of recombinant antigen was set at 100% of the positive result rate. For LOD measurement using Cultured Virus, viral supernatant was diluted in PBS, and 41.3 µL of cultured virus solution was added to sample treatment solution (550 µL) for this test. The assay was performed by adding 41.3 µL of the cultured virus solution diluted to the minimum concentration judged to be positive to the sample treatment solution (550 µL) (n=3). Based on the results of the above tests, the virus concentration at the maximum dilution at which a positive result was obtained using the cultured virus solution was used as an index of minimum detection sensitivity. For additional comparison, the swabs were also tested at exact same conditions using the previously approved SARS-CoV-2 antigen detecting rapid diagnostic kits, ESPLINE SARS-CoV-2 (Fujirebio, Tokyo, Japan) and QUICK NAVI CoV-2 Ag (Denka, Tokyo, Japan). The test was conducted according to the operation method of the kit.

For LOD measurement using Clinical Specimens, nasopharyngeal swabs used in the administrative test (real-time RT-PCR) and whose concentration (number of cycles and copy number) in real-time RT-PCR was calculated were transported from Yokohama City University Hospital and Yokohama City Institute of Public Health to the Department of Microbiology, Yokohama City University School of Medicine. All specimens were transported in virus transport medium with nasopharyngeal swabs, and the performance comparison test with the real-time RT-PCR method was performed using this kit (Rapiim SARS-CoV-2-N) and the previously approved SARS-CoV-2 antigen. The assay was performed by adding 41.3 μL of the Clinical Specimens (Pre-diluted in 3 mL of transport medium) to the sample treatment solution (550 μL).

RT-qPCR

Nasopharyngeal swab specimens from individuals confirmed with SARS-CoV-2 infection by RT-qPCR with N2 primer/probe set targeting the N gene were used for this evaluation (Shirato et al., 2020). Samples were stored in viral transport media at –80 ºC until use. Virus load in samples was estimated from cycle threshold (Ct) value. The study protocol was approved by the IRB of Yokohama City University (B200800106).

Statistical analysis

All graphs show box-and-whisker or distribution plots and approximate curves. Statistical significance was assessed by JMP 15 (SAS Institute Inc., Cary, NC, USA).
